# Supplementary material for: The interactome of intact mitochondria by cross-linking mass spectrometry provides evidence for coexisting respiratory supercomplexes
Source: Mol Cell Proteomics. 2017 Dec 8;17(2):216–32. doi: 10.1074/mcp.RA117.000470 (PMC5795388; doi:10.1074/mcp.RA117.000470)
Supplement: Supplemental Data [file supp_RA117.000470_133922_0_supp_23978_fzffwf.zip › spectra_annotation/mito_DR_spectra_annotation/408-1-2-1-5-1.pdf]

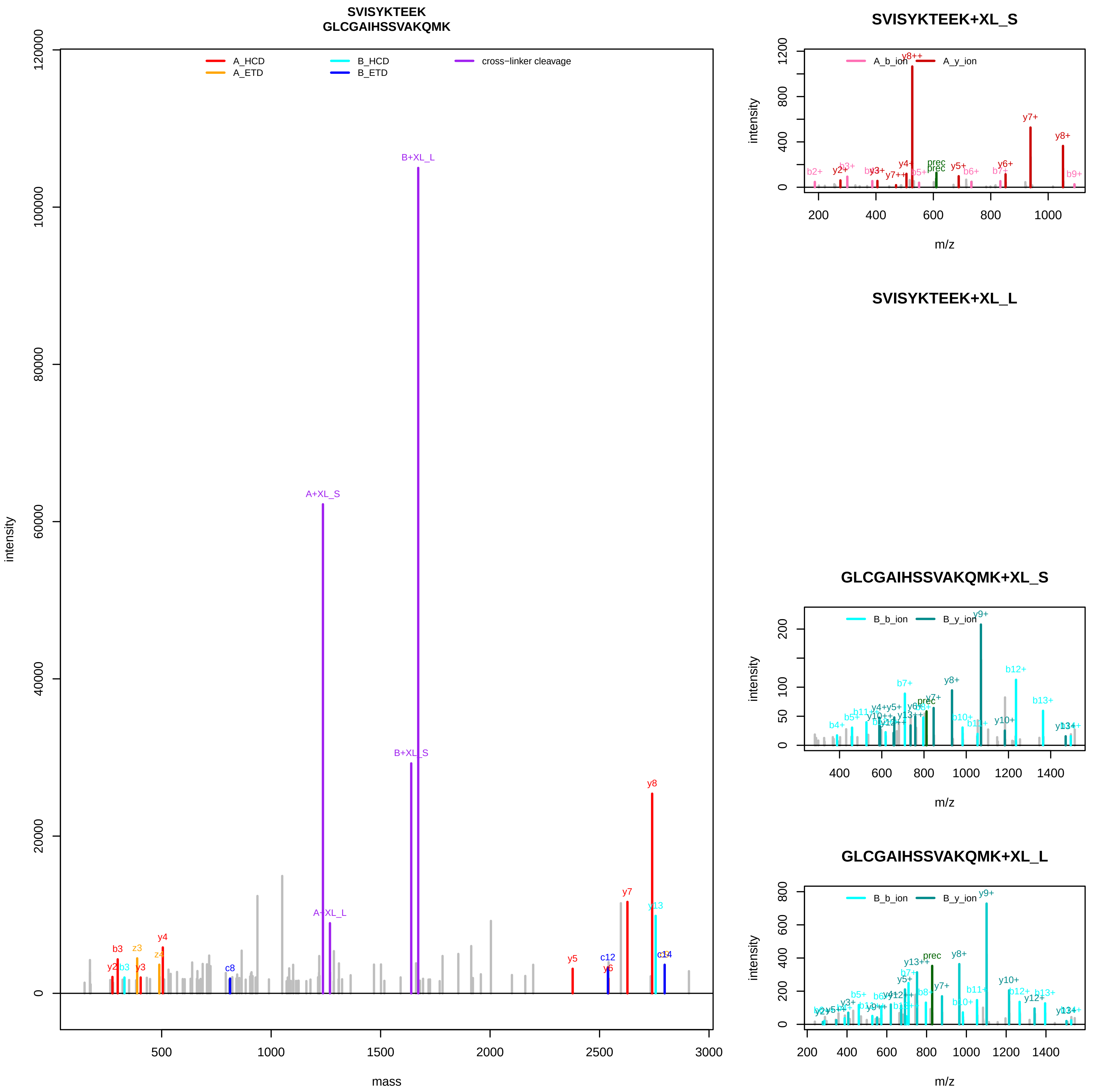
SVISYKTEEK
GLCGAIHSSVAKQMK
A_HCD
A_ETD
B_HCD
B_ETD
cross−linker cleavage
intensity
mass
0
20000
40000
60000
80000
100000
120000
500
1000
1500
2000
2500
3000
B+XL_L
A+XL_S
B+XL_S
A+XL_L
y8
y7
y13
y5
c12
y6
c14
c8
b3
y2
b3
y3
z3
z4
y4
SVISYKTEEK+XL_S
A_b_ion
A_y_ion
y8++
y7+
y8+
prec
y5+
y6+
b6+
b7+
b9+
b2+
y2+
b3+
y3+
y4+
y7++
b5+
m/z
200
400
600
800
1000
1200
SVISYKTEEK+XL_L
GLCGAIHSSVAKQMK+XL_S
B_b_ion
B_y_ion
y9+
b12+
b7+
y8+
y7+
prec
b13+
b10+
y10+
b4+
b5+
1400
GLCGAIHSSVAKQMK+XL_L
y9+
y13++
prec
y8+
b7+
y7+
b11+
y10+
b12+
b13+
y12+
b10+
b8+
y5+
b5+
y3+
b6+
y13+
